# Supplementary material for: Prolonging the circulatory half-life of C1 esterase inhibitor via albumin fusion
Source: PLoS One. 2024 Oct 23;19(10):e0305719. doi: 10.1371/journal.pone.0305719 (PMC11498661; doi:10.1371/journal.pone.0305719)
Supplement: S1 Appendix — (1) Protein sequences and lengths. The primary sequence of all recombinant proteins employed in this study are shown, along with the length in amino acids, the molecular weight in Daltons, and the calculated isoelectric point. (2) Raw data shown in Table 1. (3) Raw data shown in Fig 2B. (3) Raw data shown in Fig 3A–3D. (4) Raw data shown in Fig 3E. (5) Raw data shown in Table 2. (DOCX) [file pone.0305719.s001.docx]

**Supporting Information to Sivananthan S et al.**

1. **Protein sequences and lengths**

Below can be found the primary amino acid sequence of each recombinant C1INH- or MSA-related recombinant protein used in this study, in each case with length in amino acids, predicted molecular weight, and calculated isoelectric point (pI).

**H_6_-trC1INH(MGS)**

EAHHHHHHGSFCPGPVTLCSDLESHSTEAVLGDALVDFSLKLYHAFSAMKKVETNMAFSP

FSIASLLTQVLLGAGENTKTNLESILSYPKDFTCVHQALKGFTTKGVTSVSQIFHSPDLA

IRDTFVQASRTLYSSSPRVLSQNSDANLELINTWVAKNTNNKISRLLDSLPSDTRLVLLN

AIYLSAKWKTTFDPKKTRMEPFHFKNSVIKVPMMNSKKYPVAHFIDQTLKAKVGQLQLSH

QLSLVILVPQNLKHRLEDMEQALSPSVFKAIMEKLEMSKFQPTLLTLPRIKVTTSQDMLS

IMEKLEFFDFSYDLNLCGLTEDPDLQVSAMQHQTVLELTETGVEAAAASAISVARTLLVF

EVQQPFLFVLWDQQHKFPVFMGRVYDPRA

389 amino acids

43,697 Da

pI 7.09

**H_6_-trC1INH(MGS)-MSA**

EAHHHHHHGSFCPGPVTLCSDLESHSTEAVLGDALVDFSLKLYHAFSAMKKVETNMAFSP

FSIASLLTQVLLGAGENTKTNLESILSYPKDFTCVHQALKGFTTKGVTSVSQIFHSPDLA

IRDTFVQASRTLYSSSPRVLSQNSDANLELINTWVAKNTNNKISRLLDSLPSDTRLVLLN

AIYLSAKWKTTFDPKKTRMEPFHFKNSVIKVPMMNSKKYPVAHFIDQTLKAKVGQLQLSH

QLSLVILVPQNLKHRLEDMEQALSPSVFKAIMEKLEMSKFQPTLLTLPRIKVTTSQDMLS

IMEKLEFFDFSYDLNLCGLTEDPDLQVSAMQHQTVLELTETGVEAAAASAISVARTLLVF

EVQQPFLFVLWDQQHKFPVFMGRVYDPRAGGGGSGGGGSGGGGSHKSEIAHRYNDLGEQH

FKGLVLIAFSQYLQKCSYDEHAKLVQEVTDFAKTCVADESAANCDKSLHTLFGDKLCAIP

NLRENYGELADCCTKQEPERNECFLQHKDDNPSLPPFERPEAEAMCTSFKENPTTFMGHY

LHEVARRHPYFYAPELLYYAEQYNEILTQCCAEADKESCLTPKLDGVKEKALVSSVRQRM

KCSSMQKFGERAFKAWAVARLSQTFPNADFAEITKLATDLTKVNKECCHGDLLECADDRA

ELAKYMCENQATISSKLQTCCDKPLLKKAHCLSEVEHDTMPADLPAIAADFVEDQEVCKN

YAEAKDVFLGTFLYEYSRRHPDYSVSLLLRLAKKYEATLEKCCAEANPPACYGTVLAEFQ

PLVEEPKNLVKTNCDLYEKLGEYGFQNAILVRYTQKAPQVSTPTLVEAARNLGRVGTKCC

TLPEDQRLPCVEDYLSAILNRVCLLHEKTPVSEHVTKCCSGSLVERRPCFSALTVDETYV

PKEFKAETFTFHSDICTLPEKEKQIKKQTALAELVKHKPKATAEQLKTVMDDFAQFLDTC

CKAADKDTCFSTEGPNLVTRCKDALA

986 amino acids

110,315 Da

pI 6.00

**H_6_-MSA**

EAHHHHHHHKSEIAHRYNDLGEQHFKGLVLIAFSQYLQKCSYDEHAKLVQEVTDFAKTCV

ADESAANCDKSLHTLFGDKLCAIPNLRENYGELADCCTKQEPERNECFLQHKDDNPSLPP

FERPEAEAMCTSFKENPTTFMGHYLHEVARRHPYFYAPELLYYAEQYNEILTQCCAEADK

ESCLTPKLDGVKEKALVSSVRQRMKCSSMQKFGERAFKAWAVARLSQTFPNADFAEITKL

ATDLTKVNKECCHGDLLECADDRAELAKYMCENQATISSKLQTCCDKPLLKKAHCLSEVE

HDTMPADLPAIAADFVEDQEVCKNYAEAKDVFLGTFLYEYSRRHPDYSVSLLLRLAKKYE

ATLEKCCAEANPPACYGTVLAEFQPLVEEPKNLVKTNCDLYEKLGEYGFQNAILVRYTQK

APQVSTPTLVEAARNLGRVGTKCCTLPEDQRLPCVEDYLSAILNRVCLLHEKTPVSEHVT

KCCSGSLVERRPCFSALTVDETYVPKEFKAETFTFHSDICTLPEKEKQIKKQTALAELVK

HKPKATAEQLKTVMDDFAQFLDTCCKAADKDTCFSTEGPNLVTRCKDALA

590 amino acids

66,713 Da

pI 5.68

1. **All data from Table 1.**

| **Protein name** | **k_2_ versus Pka (X 10^4^ M^-1^s^-1^)** | **k_2_ versus C1s (X 10^4^ M^-1^s^-1^)** | **SI versus Pka** |
| --- | --- | --- | --- |
| Trial 1 | 2.35 | 5.3 | 3.3 |
| Trial 2 | 2.25 | 5.6 | 3.3 |
| Trial 3 | 2.00 | 6.3 | 3.2 |
| Trial 4 | 2.45 | - | 3.3 |
| Trial 5 | 2.55 | - | 3.2 |
| pdC1INH average | 2.3 ± 0.2*** | 5.7 ± 0.5 | 3.3 ± 0.06***^^^ |
| Trial 1 | 2.00 | 4.3 | 5.3 |
| Trial 2 | 2.10 | 4.7 | 5.8 |
| Trial 3 | 1.95 | 5.7 | 5.2 |
| Trial 4 | 1.95 | - | 5.1 |
| Trial 5 | 2.05 | - | 5.5 |
| H_6_-trC1INH(MGS) average | 2.01 ± 0.07*** | 5.0 ± 0.5 | 5.3 ± 0.3 |
| Trial 1 | 1.2 | 4.68 | 7.0 |
| Trial 2 | 1.25 | 5.42 | 6.9 |
| Trial 3 | 1.35 | 5.22 | 6.7 |
| Trial 4 | 1.2 |  | 6.8 |
| Trial 5 | 1.35 |  | 7.1 |
| H_6_-trC1INH(MGS)-MSA average | 1.3 ± 0.08 | 5.1 ± 0.4 | 6.9 ± 0.1 |
| Results are the mean of 5 determinations (SI or k_2_ for kallikrein), ± SD, or 3 determinations (k_2_ for C1s). ***, p < 0.001 versus H_6_-trC1INH(MGS)-MSA. ^^^, p < 0.001 versus H_6_-trC1INH(MGS). Each trial is shown. | | | |

1. **All data from Figure 2B**

1. **All data from Figure 3A, 3B, 3C, and 3D**

1. All data from Figure 3E

|  | **H6-trC1INH(MGS)-MSA** | | **H6-MSA** | | |  |  |  |
| --- | --- | --- | --- | --- | --- | --- | --- | --- |
| **Time (hours after injection)** | **16** | **18** | **24** | **28** | |  |  |  |
| Mouse 1 | 20.5 | 17.1 | 32.5 | 22.6 | |  |  |  |
| Mouse 2 | 25.6 | 21.0 | 32.2 | 21.0 | |  |  |  |
| Mouse 3 | 22.6 | 18.9 | 31.1 | 22.0 | |  |  |  |
| Mouse 4 | 25.7 | 20.6 | 31.8 | 22.0 | |  |  |  |
| Mouse 5 | 23.2 | 18.7 | 31.5 | 22.8 | |  |  |  |
| Mouse 6 | 24.8 | 19.4 | 31.4 | 21.8 | |  |  |  |
|  | | | | |  | |  |  |
|  | | | | |  | |  |  |
|  | | | | |  | |  |  |
|  | | | | |  | |  |  |
|  | | | | |  | |  |  |
|  | | | | |  | |  |  |
|  | | | | |  | |  |  |

1. **All data from Table 2**

| **Protein name** | **Terminal half-life (hours)** | **Area Under the observed Curve (AUC; %-hours)** |
| --- | --- | --- |
| Trial 1 | 5.3 | 81 |
| Trial 2 | 8.1 | 68 |
| Trial 3 | 3.1 | 93 |
| Trial 4 | 3.1 | 64 |
| Trial 5 | 5.1 | 104 |
| Trial 6 | 3.3 | 84 |
| H_6_-trC1INH(MGS) average | 5 ± 2 | 80 ± 20 |
| Trial 1 | 12.9 | 303 |
| Trial 2 | 20.2 | 323 |
| Trial 3 | 10.8 | 298 |
| Trial 4 | 11.7 | 288 |
| Trial 5 | 14.5 | 348 |
| Trial 6 | 14.4 | 313 |
| H_6_-trC1INH(MGS)-MSA average | 14 ± 3* | 310 ± 20*** |
| Trial 1 | 21.6 | 340 |
| Trial 2 | 35.9 | 327 |
| Trial 3 | 13.3 | 344 |
| Trial 4 | 18.3 | 330 |
| Trial 5 | 24.2 | 338 |
| Trial 6 | 15.1 | 349 |
| H_6_-MSA average | 21 ± 8*** | 340 ± 8*** |
| Results are the mean of 6 determinations, ± SD. *, p < 0.05; *******, p < 0.001 versus H_6_-trC1INH(MGS). | | |

1. **All data relating to effect of sex or weight on terminal half-life, by protein**

|  | **Half-life values by injected protein and sex** | | | |  |  |
| --- | --- | --- | --- | --- | --- | --- |
|  | **H6-trC1INH(MGS)** | | **H6-trC1INH(MGS)-MSA** | | **H6-MSA** |  |
|  | Male | Female | Male | Female | Male | Female |
|  | 0.338693 | 0.219536 | 1.907285 | 1.531063 | 4.014926 | 2.587195 |
|  | 0.129881 | 0.127471 | 1.497898 | 1.997685 | 2.70063 | 2.602597 |
|  | 0.210745 | 0.138842 | 1.754144 | 1.759024 | 3.106161 | 2.600269 |
|  |  |  |  |  |  |  |
| Average | 0.22644 | 0.16195 | 1.7197757 | 1.76259067 | 3.273906 | 2.596687 |
| SD | 0.105287 | 0.050194 | 0.2068461 | 0.23333145 | 0.673014 | 0.008302 |
|  |  |  |  |  |  |  |
|  | **Half-life values by injected protein and weight (stratified, 2 groups)** | | | | | |
|  | **H6-trC1INH(MGS)** | | **H6-trC1INH(MGS)-MSA** | | **H6-MSA** |  |
|  | Heavier | Lighter | Heavier | Lighter | Heavier | Lighter |
|  | 0.338693 | 0.219536 | 1.531063 | 1.907285 | 4.014926 | 2.587195 |
|  | 0.129881 | 0.210745 | 1.997685 | 1.497898 | 2.70063 | 3.106161 |
|  | 0.127471 | 0.138842 | 1.754144 | 1.759024 | 2.602597 | 2.600269 |
|  |  |  |  |  |  |  |
| Average | 0.198682 | 0.189708 | 1.760964 | 1.72140233 | 3.106051 | 2.764542 |
| SD | 0.121259 | 0.04427 | 0.2333857 | 0.20727029 | 0.788634 | 0.295923 |
|  |  |  |  |  |  |  |

1. **All data relating to mouse weight**

|  | **Weight of mice whose data is shown in Fig 3 A-D** | | |
| --- | --- | --- | --- |
|  | H6-trC1INH(MGS) | H6-trC1INH(MGS)-MSA | H6-MSA |
|  |  |  |  |
|  | 26.6 | 28 | 24.4 |
|  | 33.8 | 29 | 41.6 |
|  | 28.4 | 31 | 34.8 |
|  | 29.2 | 32 | 31 |
|  | 28 | 40.6 | 25.8 |
|  | 27.8 | 25 | 26.6 |
|  |  |  |  |
| Average | 28.96666667 | 30.93333333 | 30.7 |
| SD | 2.515286597 | 5.331666406 | 6.576017 |
|  |  |  |  |
|  | **Weight of mice whose data is shown in Fig 3 E** | |  |
|  |  | H6-trC1INH(MGS)-MSA | H6-MSA |
|  |  | 34 | 30 |
|  |  | 31.4 | 33 |
|  |  | 35.6 | 27.8 |
|  |  | 29 | 30 |
|  |  | 29.5 | 34.8 |
|  |  | 27.8 | 31.4 |
|  |  |  |  |
| Average |  | 31.21666667 | 31.16667 |
| SD |  | 3.050519082 | 2.476826 |

|  | **Weight of all mice (n=30)** | |
| --- | --- | --- |
|  |  |  |
|  | 26.6 |  |
|  | 33.8 |  |
|  | 28.4 |  |
|  | 29.2 |  |
|  | 28 |  |
|  | 27.8 |  |
|  | 28 |  |
|  | 29 |  |
|  | 31 |  |
|  | 32 |  |
|  | 40.6 |  |
|  | 25 |  |
|  | 24.4 |  |
|  | 41.6 |  |
|  | 34.8 |  |
|  | 31 |  |
|  | 25.8 |  |
|  | 26.6 |  |
|  | 34 |  |
|  | 31.4 |  |
|  | 35.6 |  |
|  | 29 |  |
|  | 29.5 |  |
|  | 27.8 |  |
|  | 30 |  |
|  | 33 |  |
|  | 27.8 |  |
|  | 30 |  |
|  | 34.8 |  |
|  | 31.4 |  |
|  |  |  |
| Average | 30.73448276 |  |
| SD | 4.102689054 |  |
