# Supplementary material for: Prolonging the circulatory half-life of C1 esterase inhibitor via albumin fusion
Source: PLoS One. 2024 Oct 23;19(10):e0305719. doi: 10.1371/journal.pone.0305719 (PMC11498661; doi:10.1371/journal.pone.0305719)
Supplement: S2 Appendix — (PDF) [file pone.0305719.s002.pdf]

S2 Appendix (raw gel and blot images)

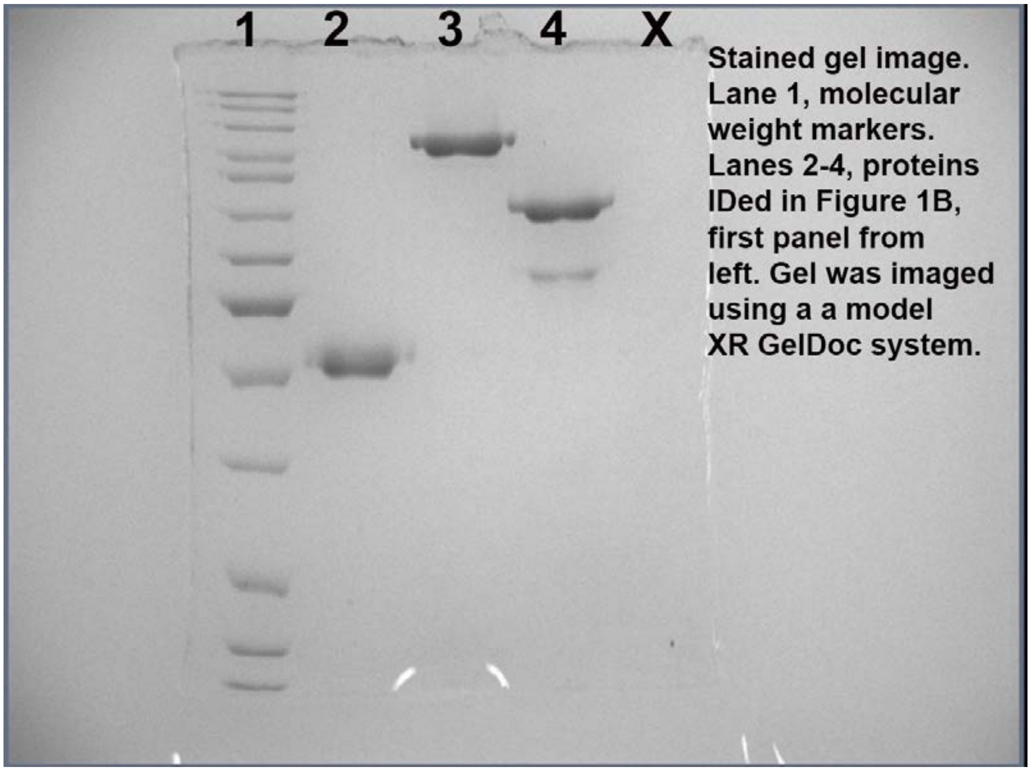

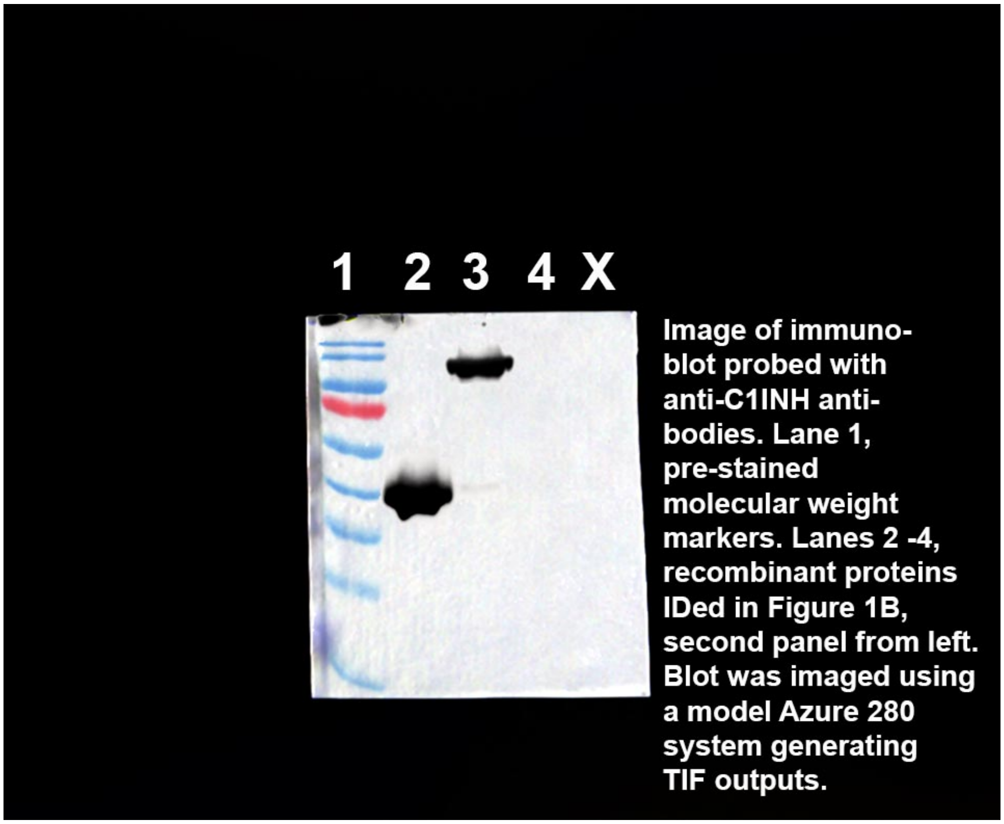

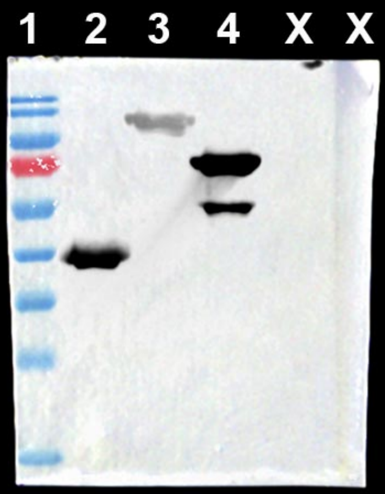

Image of immuno-blot probed with anti-hexahistidine antibodies. Lane 1 pre-stained molecular weight markers. Lanes 2-4 recombinant proteins IDed in Figure 1B, third panel from left. Blot was imaged using a model Azure 280 system generatingTIF format outputs.

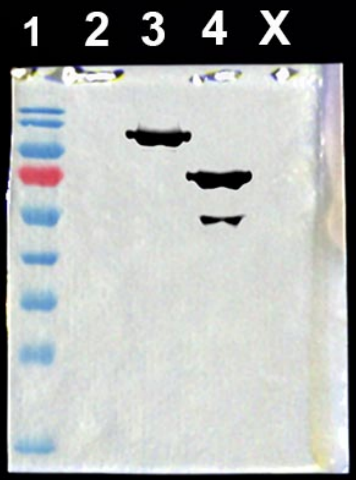

Image of blot probed with anti-MSA anti-bodies. Lane 1, pre-stained molecular weight markers. Lanes 2-4, recomb-inant proteins IDed in Figure 1B, fourth panel from left. Image was generated using a model Azure 280 system generating TIF format outputs.

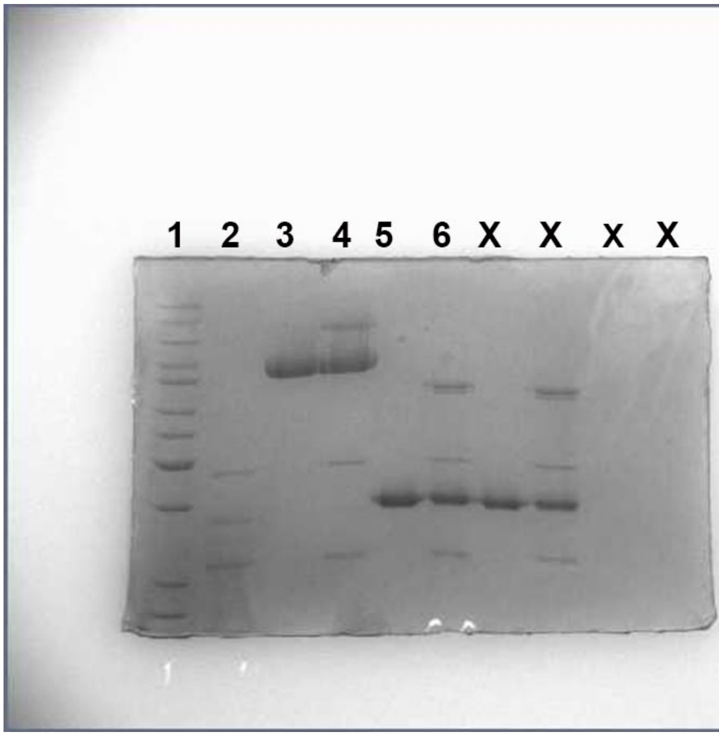

Stained gel image. Lane 1 molecular weight markers. Lanes 2-6 proteins ID-ed in Figure 2Ai Gel was imaged using an model Azure 280 system generating TIF format outputs

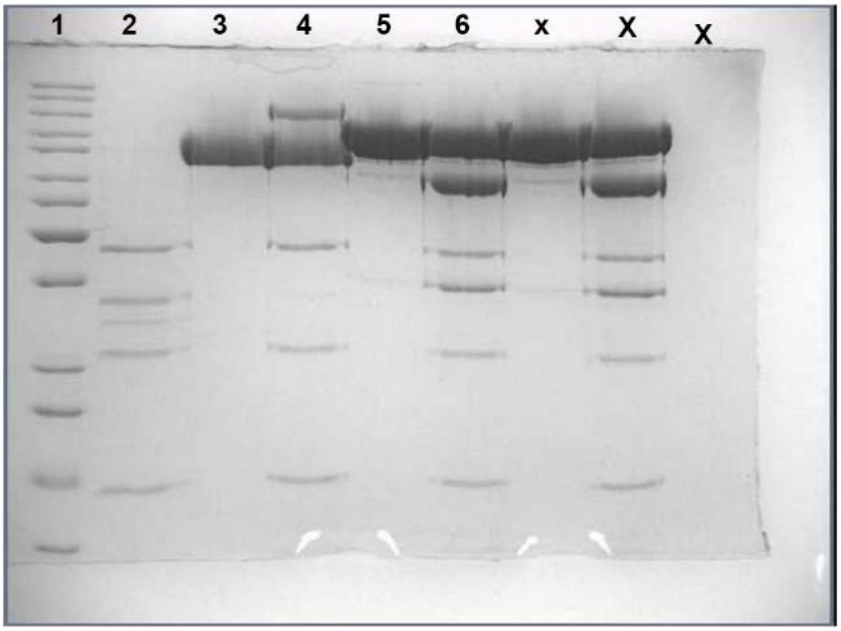

Image of stained gel is shown. Lane 1, molecular weight markers. Lanes 2-6 are proteins IDed in Figure 2aii. Gel was imaged using a model XR GelDoc system generating TIF file format outputs.
