# Supplementary material for: Prolonging the circulatory half-life of C1 esterase inhibitor via albumin fusion
Source: PLoS One. 2024 Oct 23;19(10):e0305719. doi: 10.1371/journal.pone.0305719 (PMC11498661; doi:10.1371/journal.pone.0305719)
Supplement: Attachment — Submitted filename: Comments_PLOS ONE.docx [file pone.0305719.s003.docx]

**Journal:** PLOS ONE

**Manuscript Number:** PONE-D-24-10397

**Article Type:** Research Article

**Title:** Prolonging the circulatory half-life of C1 esterase inhibitor via albumin fusion

**Recommendation:** Publish after minor revisions are noted.

**Comments:**

The manuscript by Sivananthan and colleagues described the Hereditary Angioedema (HAE) an autosomal dominant episodic swelling disease occur in various body parts due to deficiency in C1-esterase inhibitor (C1INH), a regulator of several proteases eg. Plasma Kallikrein (Pka). Till date, many C1INH treatments have been employed to prevent this disease yet exhibit short circulatory half-life. In this study, they designed, expressed and purified recombinant proteins H6-trC1INH(MGS), H6-trC1INH(MGS)-MSA, and H6-MSA using Pichia pastoris. They found the significant extended circulatory half-life of recombinant H6-trC1INH(MGS)-MSA compared to H6-trC1INH(MGS).

Author also tried to perform vivo study using the CDI mice in which they injected the N-glycosylated C1INH recombinant protein with /or without albumin fusion, separately. They found the mean terminal half-life of H6-trC1INH(MGS)-MSA was 3-fold higher with reduction in Pka inhibition than that of wildtype H6-trC1INH(MGS). Suggesting that H6-trC1INH(MGS)-MSA discovery is advantage in mice model of HRE.

Overall, this an interesting manuscript showing extended circulatory half-life of N-glycosylated C1INH recombinant protein via albumin fusion.

**Comment 1:** Please add the catalog number for all purchased reagents. For examples: Antibodies, Pka, Complement component.

**Comment 2:** What is the rationale for dose determination of N-glycosylated C1INH recombinant proteins injected to mice model?

**Comment 3:** Please see the page 15, line 287,H6-trC1INH(MGS)-MSA or H6-MSA (90±4% and 95%±3%, respectively.

**Comment 4:** Please tale a look on Y-axis scale of figure 3A. It looks to weird. There should be 50% residual protein in the middle of y axis scale instead of 10%. Please correct it accordingly.
